# Controlled Fragmentation of Urinary Stones as a Method of Preventing Inflammatory Infections in the Treatment of Urolithiasis (Experience in Successful Clinical Use)

**DOI:** 10.17691/stm2021.13.3.07

**Published:** 2021-06-28

**Authors:** O.S. Streltsova, V.V. Vlasov, E.V. Grebenkin, A.E. Antonyan, V.V. Elagin, V.F. Lazukin, N.I. Ignatova, V.A. Kamensky

**Affiliations:** Professor, Е.V. Shakhov Urology Department; Privolzhsky Research Medical University, 10/1 Minin and Pozharsky Square, Nizhny Novgorod, 603005, Russia; Associate Professor, Е.V. Shakhov Urology Department; Privolzhsky Research Medical University, 10/1 Minin and Pozharsky Square, Nizhny Novgorod, 603005, Russia; PhD Student, Е.V. Shakhov Urology Department; Privolzhsky Research Medical University, 10/1 Minin and Pozharsky Square, Nizhny Novgorod, 603005, Russia; PhD Student, Е.V. Shakhov Urology Department; Privolzhsky Research Medical University, 10/1 Minin and Pozharsky Square, Nizhny Novgorod, 603005, Russia; Researcher, Research Institute of Experimental Oncology and Biomedical Technologies; Privolzhsky Research Medical University, 10/1 Minin and Pozharsky Square, Nizhny Novgorod, 603005, Russia; Associate Professor, Department of Medical Physics and Informatics; Privolzhsky Research Medical University, 10/1 Minin and Pozharsky Square, Nizhny Novgorod, 603005, Russia; Associate Professor, Department of Epidemiology, Microbiology and Evidence-Based Medicine; Privolzhsky Research Medical University, 10/1 Minin and Pozharsky Square, Nizhny Novgorod, 603005, Russia; Leading Researcher, Federal Research Center Institute of Applied Physics of the Russian Academy of Sciences, 46 Ulyanova St., Nizhny Novgorod, 603950, Russia

**Keywords:** urolithiasis, infectious and inflammatory complications of lithotripsy, pyelonephritis, stone fragmentation in urolithiasis

## Abstract

The introduction of technologically advanced methods of lithotripsy into medical practice changes the nature of postoperative complications. Among them, the main complications are inflammatory infections. This largely determines the search for new, improved methods of stone fragmentation avoiding small stone fragments and dissemination of the pelvicalyceal system of the kidney with stone-associated infection. The authors have developed a method for controlled stone fragmentation using a continuous-wave diode laser with a hot-spot effect at the optical fiber end.

**The aim of the study** was to evaluate the efficacy of controlled urinary stone fragmentation using a continuous-wave diode laser with a highly heated distal end of the optical fiber light guide as a method of preventing inflammatory infections in clinical practice.

**Materials and Methods:**

We analyzed 1666 case histories of urolithiasis patients who underwent percutaneous nephrolithotripsy/ nephrolithoextraction and contact ureterolithotripsy/ureteroextraction, we also performed a prospective analysis of complications based on the Clavien–Dindo classification in 90 patients who underwent fine fragmentation of stones with various lithotripters: ultrasonic, pneumatic, and holmium laser. The method of controlled stone fragmentation by a diode laser with a hot-spot effect was tested on postoperative samples of 26 renal calculi. For the first time in clinical practice, this method was tested in the bladder cavity (n=10).

**Results:**

In the percutaneous nephrolithotripsy group, postoperative infectious and inflammatory complications occurred in 34.1% of cases, in the percutaneous nephrolithoextraction group — in 24.6%, in the contact ureterolithotripsy group — in 7.8%, in the ureterolithoextraction group — in 2.5%. The analysis made it possible to identify factors promoting the development of infectious and inflammatory complications. For the first time in clinical practice, there were successfully performed ten operations of stone fragmentation using a continuous-wave diode laser with a hot-spot effect. Controlled coarse fragmentation of stones providing the possibility to reduce the number of infectious and inflammatory complications was performed in the bladder as a model for testing the method.

**Conclusion:**

The method of laser-induced controlled coarse fragmentation of stones with a hot-spot effect, developed and tested in clinical practice, is promising for the prevention of infectious and inflammatory complications in patients with potentially infected stones since their fine fragmentation and, consequently, spread of stone-associated toxins and microflora within the urinary system is avoided.

## Introduction

The incidence and prevalence of urolithiasis in the world are growing from one year to another [1, 2]. Percutaneous urolithiasis surgery has been used and improved for about 40 years. Today, percutaneous nephrolithotripsy is the method of choice in the surgical treatment of patients with kidney stones. There are several types of contact lithotripters: electrohydraulic, ultrasonic, pneumatic, electrokinetic, laser. Each method has its own advantages and disadvantages. Laser lithotripsy allowing fragmentation of even most dense stones both in the ureter and in the kidney is recognized as a universal method. However, the introduction of high-tech interventions into medical practice has led to changes in the nature of postoperative complications. Inflammatory infections and the presence of residual lithiasis have become the main complications [3–5]. According to the literature [6], 10–15% of urinary stones have infectious origin, according to other sources [7], the prevalence of stones associated with an infectious agent amounts to 30%.

All currently presented lithotripsy methods are aimed at fine fragmentation of the stone mass (dusting mechanism). However, the structures remaining in the renal system can cause development of postoperative inflammatory complications, and the fragments as crystallization centers disrupting the colloidal system of urine are able to cause recurrence of stone formation. It is known [8] that mechanical injury to the renal parenchyma during percutaneous access to the renal pelvis and stone fragmentation during lithotripsy can trigger the growth of microorganisms integrated into the stone-associated biofilm. Migration of microorganisms and their toxins into the tissues and vascular bed of the kidney can lead to the development of pyelonephritis, systemic inflammatory response syndrome, and sepsis. In addition, bacteria in the biofilm are able to stay in kidney stones for a long time. The ability to form biofilms has been found in more than 50 species of bacteria [9]. Thus, bacterial associations and mechanical injury to the pelvic tissue by a stone are the reasons for the development of inflammation in the area of stone location.

Antibiotic resistance, the recent growth of which has been noted by many authors [10, 11] aggravates the risk of systemic inflammatory response with the subsequent development of septic complications including endotoxic shock, therefore antibiotic therapy is unsuccessful in some cases.

All of the above determines the need to find new methods of renal cavity system sanitation and new methods of lithotripsy. Previously, the authors developed a method of controlled stone fragmentation using a continuous-wave diode laser with a hot-spot effect at the optical fiber end. It is based on the use of optical radiation at the optical fiber end equipped with a highly absorbing coating based on graphite powder and silicone varnish. Experimental studies carried out by our group have shown that the method has a bactericidal effect against *Escherichia coli*. The temperature effect, as well as the effect of thermal turbulent fluid flows and, supposedly, cavitating bubbles, play the main role in the process [12]. However, it turned out to be difficult to apply the method in clinical practice due to limitations associated with the mineralogical composition of calculi, and due to the lack of a technique for creating perforated channels along the planned fragmentation line in the stone mass. At present, studies have been carried out that made it possible to determine the modes of laser exposure providing fragmentation of all calculi without scattering the microbial contents of stones all over the urinary system and exerting a bactericidal effect in the stone fracture zone during fragmentation. That was a way to prevent inflammatory infections and allowed introducing the method of controlled fragmentation of infected stones into clinical practice [13, 14].

**The aim of the study** was to perform clinical testing of controlled urinary stone fragmentation using a continuous-wave diode laser with a highly heated distal end of the optic fiber light guide as a method of preventing inflammatory infections.

## Materials and Methods

We performed a retrospective analysis of 1666 case histories of patients with urolithiasis who underwent percutaneous nephrolithotripsy/extraction and contact ureterolithotripsy/extraction at the urology clinic of Privolzhsky Research Medical University based at N.A. Semashko Nizhny Novgorod Regional Clinical Hospital (Russia). Of them, 361 patients underwent percutaneous nephrolithotripsy, percutaneous nephrolithoextraction was performed in 240 patients, contact ureterolithotripsy — in 294 patients, ureterolithoextraction — in 771 patients. All patients were individuals who reached the age of 18 and gave written consent to perform the surgery. Their average age was 54.0±2.3 years. Surgical interventions were performed according to medical indications. Based on the results of laboratory studies of blood and urine, assessment of the temperature curve, and clinical picture analysis, a group of patients (n=224; 13.4±0.9%) was identified who developed infectious and inflammatory complications characterized by fever and/or inflammatory changes according to general blood and urine analyses. There were 69 males (30.8±3.1%), 155 females (69.2±3.1%).

The study complies with the Declaration of Helsinki (2013). Written informed consent was obtained from every patient.

The results were statistically processed using the Statistica 10.0 software package. The presence of statistically significant differences between the comparison groups represented by fractions (frequencies) of the feature of interest was determined using Fisher’s angular transformation [15], where φ=2**·**arcsin(*p*^½^); *p* is the frequency of feature manifestation, expressed in fractions of a unit. Differences were considered statistically significant at p<0.05.

The standard deviation of the percentage (σ_р_%) was calculated by the formula

$$\sigma_{p}\%=\sqrt{\frac{p(1=p)}{n}},$$

where p is the percentage; σ_р_% is the standard deviation of the percentage; *n* is the total number of elements in the sample.

To identify the factors promoting the development of infectious and inflammatory complications (fine fragmentation of stones and intrapelvic pressure), there was performed a prospective analysis of postoperative complications based on the Clavien–Dindo classification of surgical complications [16] in three groups of patients (n=30 in each). All of them underwent percutaneous nephrolithotripsy for calculi of the upper urinary tract with the use of techniques involving fine fragmentation of stones with pneumatic, ultrasonic lithotripters, and holmium laser.

To assess the effect of intrapelvic pressure during surgery, the Omron medical tonometer (Japan) modified to measure intrapelvic pressure was used in these patients. It was connected to a ureteral catheter through a sterile adapter made on the basis of an intravenous infusion system. The intrapelvic pressure was assessed during the entire period of lithotripsy. All stones have been subjected to mineralogical analysis.

To prevent fine fragmentation, the authors have developed a fragmentation method using an available diode laser with a highly heated distal end of the optical fiber light guide.

**Figure 1:**
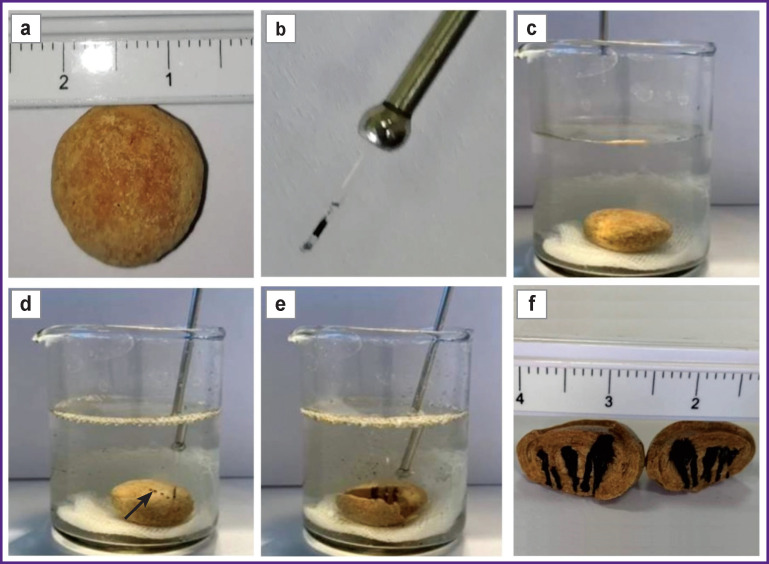
Stages of controlled stone fragmentation *in vitro*: (a) measurement of the stone size; (b) robe-pointed bougie with an optical fiber inserted into it; the distal end of the fiber coated with a layer of a colloidal solution of graphite microparticles in a silicon varnish; (c) the stone is placed in a container with a saline solution; (d) perforated channels in the stone (the line of the future fracture is indicated by the arrow); (e) fracture of the stone; (f) perforated channels in fragmented stone

The introduction of an optical fiber into a hollow guide tube (a long probe-pointed bougie) or into a ureteral catheter (6 Ch), from which the distal end was cut off, made it possible to start performing operations in the clinic. It was necessary to install a rubber restrictor at the proximal end of the optical fiber, which did not allow the distal end of the optical fiber larger than the stone thickness as determined by ultrasound or CT to be pulled out of the hollow tube of the guidewire/ureteral catheter. This way, the possibility of moving the end face of the optical fiber outside the stone was avoided. The probe with the optical fiber was intimately pressed against the stone perpendicular to its surface along the line of the planned fracture and the pulse was activated. Several perforation channels were created along the planned fracture line (Figure 1).

To test the technology of controlled fragmentation *in vitro*, we used postoperative samples (26 in total) of renal calculi ranging in size from 10 to 21 mm with different X-ray densities in Hounsfield units (127–1433 HU).

**Figure 2:**
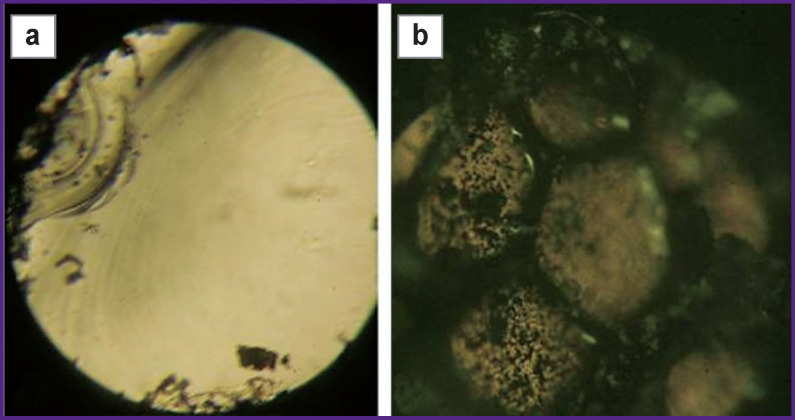
The output end of the quartz fiber light guide without blackening (a) and with blackening (b); optical fiber diameter is 550 μm

A LAKHTA-MILON diode laser (MILON Laser, Russia) with a wavelength of 1470 nm, a power of 10 W, certified for use in medicine, was applied as a contact lithotripter. This laser is characterized by continuous generation of laser radiation, which allows “non-explosive” calculus fragmentation, i.e. fragmentation without forming small fragments. The approach to controlled fragmentation is based on the initiation principle, which implies the use of a highly absorbing coating at the working end of an optical fiber as a factor increasing radiation absorption coefficient [17]. The layer of a highly absorbing coating was formed using the method of Alta® Modular Laser System (USA). For this, special carbon-containing tablets were used (Dental Photonics, Inc., USA). A colloidal solution of graphite microparticles in silicon varnish forming the heat- and wear-resistant layer at the end of the optical fiber and allowing the “hot-spot” method to be implemented [18] was also used as a highly absorbing coating. It is important to note that with this approach, radiation does not affect the object itself, but the converting element at the end of the optical fiber (in our case, it is a layer of carbon or graphite microparticles in silicon varnish, Figure 2), which contacts the object. Controlled fragmentation is achieved due to thermal action (the temperature near the end face of the optical fiber is about 2000 K) of continuous-wave laser radiation and subsequent mechanical cracking of the calculus. The technical result of coarse fragmentation was achieved by using the mode of carbonization of the stone substance by the high temperature of the fiber end. The criterion for success was breaking the stone into two fragments.

The clinical study of applicability of the “hot-spot” method with the use of continuous-wave diode laser radiation for urinary stone fragmentation in patients was approved by the Ethics Committee of Privolzhsky Research Medical University (Russia).

The technology was first implemented on a bladder as a model for controlled coarse fragmentation in the operating room setting.

## Results

**Figure 3:**
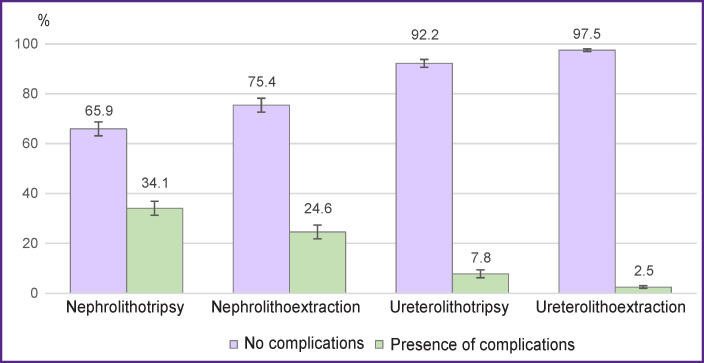
Distribution of patients with postoperative infectious and inflammatory complications, depending on the type of endoscopic treatment of urolithiasis

On the basis of the retrospective analysis for the presence of postoperative inflammatory diseases in the selected case histories of 1666 patients, a group of individuals (n=224; 13.4±0.9%) was identified who had infectious and inflammatory complications characterized by fever and/or inflammatory changes in general blood and urine tests (Figure 3). It was found that in the group of percutaneous nephrolithotripsy, postoperative infectious and inflammatory complications occurred in 34.1±2.5% of cases (123/361), in the group of percutaneous nephrolithoextraction — in 24.6±2.8% of cases (59/240). In the group of operations on the ureters, the proportion of patients with postoperative infectious and inflammatory complications after contact ureterolithotripsy was 7.8±1.6% of cases (23/294), and after ureterolithoextraction — 2.5±0.6% of cases (19/771).

Table 1 shows the significance levels (p) of differences in the incidence of complications in different types of endoscopic treatment of urolithiasis calculated using Fisher’s angular transformation.

**Table 1:** Significance levels of differences in the incidence of complications for different types of endoscopic treatment

Type of treatment	Nephrolithoextraction	Ureterolithotripsy	Ureterolithoextraction
Nephrolithotripsy	<0.01	<0.001	<0.001
Nephrolithoextraction	—	<0.001	<0.001
Ureterolithotripsy	<0.001	—	<0.001

It follows from Table 1 that there is a statistically significant difference between the incidence rates of complications at p<0.001 for all the endoscopic treatment methods applied. The exception is a couple of methods (nephrolithotripsy and nephrolithoextraction), where the level of significance of differences was p<0.01. The data obtained indicate that in terms of infectious and inflammatory complications occurring in the postoperative period after endoscopic treatment of urolithiasis, nephrolithotripsy is the least safe method, while ureterolithoextraction is the safest one.

According to the bacteriological analysis of urine, 59.8±3.3% of patients in the selected group (134/224) had positive outcomes in the preoperative period. The spectrum of the isolated flora was represented by *Escherichia coli* (29%), *Enterobacter agglomerans* (24%), *Staphylococcus epidermidis* (20%), *Enterococcus faecalis* (15%), *Proteus mirabilis* (4%), *Pseudomonas aeruginosa* (3%), *Staphylococcus saprophyticus* (3%), *Enterobacter gergoviae* (1%), *Citrobacter spp*. (1%). All patients received preoperative preventive therapy with antibiotics (cephalosporin drugs) and treatment.

According to the results of analysis, 44.6±8.3% (100/224) of the examined patients in the group with postoperative infectious and inflammatory complications developed a systemic inflammatory response syndrome. According to the ASCP/SCCM criteria (1992), it was diagnosed in the presence of two or more of the following symptoms: temperature ≥38°C or ≤36°C; heart rate ≥90 per minute; RR>20 per minute or hyperventilation (PaCO_2_≤32 mm Hg); blood leukocytes >12**·**10^9^/ml or <4**·**10^9^/ml or immature forms more than 10%. Notably, the largest number of systemic inflammatory response syndrome cases was observed in the group of percutaneous nephrolithotripsy — in 20.8±2.1% (75/361) of patients. There was no such complication recorded in the ureterolithoextraction group. In the postoperative period, sepsis developed in 2 patients (2/361) from the percutaneous nephrolithotripsy group, 1 patient (1/240) from the percutaneous nephrolithotripsy group, and 1 patient (1/294) from the contact ureterolithotripsy group. Postoperative febrile fever occurred in all patients with calculi size ≥6 cm (11.16%; 25/224). Subfebrile fever was observed in other 37.5±6.3% (84/224) of patients. It was noted that leukocyturia was detected in the postoperative general urine analysis of 70.0±3.1% (157/224) patients with fever.

In 21 out of 224 cases, it was required to perform a puncture nephrostomy or ureteral stenting in connection with inflammatory response and functional obstruction of the upper urinary tract in the postoperative period. Nephrectomy was performed in this hospitalization following the developed purulent-destructive pyelonephritis in 2 cases after percutaneous nephrolithoextraction, in 2 cases after percutaneous nephrolithotripsy, and in 1 case in connection with bleeding after percutaneous nephrolithotripsy.

A prospective analysis of postoperative complications in patients who underwent lithotripsy with three fine fragmentation techniques showed the presence of postoperative infectious and inflammatory complications (temperature reaction, pyelonephritis) in 36.7% of cases (11/30) in the pneumatic lithotripter group and in the holmium laser group, as well as in 40% of cases (12/30) in the ultrasonic lithotripsy group. Notably, there were no statistically significant differences in gender and age characteristics, body mass index, localization of stones, X-ray density of calculi in Hounsfield units, according to CT, laboratory blood and urine tests (p<0.05). It was found that, regardless of the applied lithotripsy technique, episodes of elevated intrapelvic pressure above 30 mm Hg were observed, which served as a factor contributing to the development of infectious and inflammatory complications due to pyelotubular and pyelovenous refluxes. In the pneumatic lithotripter group, this index was 32.5±3.9 mm Hg, in the ultrasonic lithotripter group — 31.3±4.0 mm Hg, in the holmium laser group — 33.2±5.3 mm Hg. There were no statistically significant differences in the duration of surgery in the three groups (p<0.05). The contingency coefficient of the relationship between infectious and inflammatory complications in the postoperative period and intraoperative intrapelvic pressure elevation above 30 mm Hg confirmed their interrelation (Table 2).

**Table 2:** Relationship between postoperative infectious and inflammatory complications and intraoperative intrapelvic pressure elevation above 30 mm Hg

Method	Contingency coefficient	Factor influence
Pneumatic lithotripter	0.496	Present
Ultrasonic lithotripter	0.609	Present
Holmium laser	0.433	Present

Patients with phosphate and phosphate-containing calculi, which occurred in 43 cases out of 90, were found to develop infectious and inflammatory complications more often — in 79.1% of cases (34/43). When there were urate calculi (15 out of 90 were identified) postoperative infectious and inflammatory complications occurred only in 2 patients — 13.3% (2/15). In the presence of oxalate and mixed phosphate-free calculi, there were no infectious and inflammatory complications recorded in the postoperative period. Therefore, a statistically significant relationship was established between the presence of phosphates in the calculus and the occurrence of infectious and inflammatory complications in all three study groups of patients (p<0.05).

Thus, the retrospective and prospective studies made it possible to establish that fine-piece fragmentation of infected stones (most often these were phosphate-containing stones containing an organic component, microflora, and toxins) and intrapelvic pressure elevation causing pelvic-tubular and pelvic-venous reflux are risk factors for the development of postoperative infectious and inflammatory complications in the kidneys.

When performing controlled fragmentation, the use of a guide tube makes it easy to remove the optical fiber from the stone mass and begin to form a new channel. The fragmentation time of stones *in vitro* ranges from 2 to 100 s. Out of 26 fragmented stones used for testing and developing the technology, small fragments of 2–3 mm in the number of up to 5 pieces were formed in 7 cases (26.9%).

**Figure 4:**
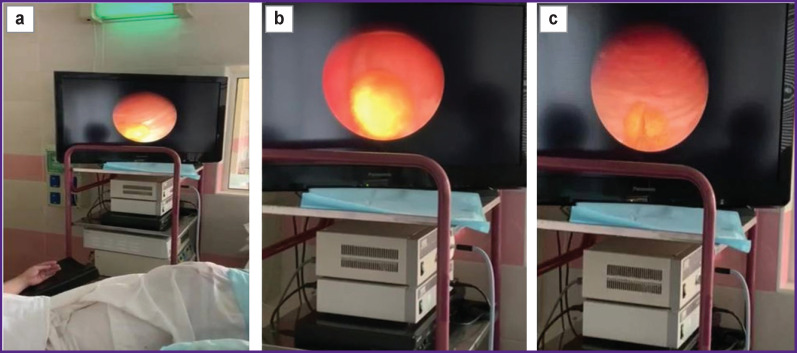
Controlled stone fragmentation in the operating room: (a), (b) stones in the urinary bladder cavity; (c) fracture of the stone in the urinary bladder cavity

The urinary bladder was chosen for testing stone fragmentation in the urinary system. There were performed 10 endoscopic operations. The stones had an X-ray CT density of up to 1000 HU. The predetermined success criterion of breaking the stone into 2 fragments was achieved in all cases (Figure 4).

The fragments were removed with forceps or they were subjected to further fragmentation (if necessary). In the fragmentation process, there was no need to reapply a highly absorbing coating on the working end of the optical fiber as the high temperature at the working end triggered carbonization of organic inclusions in the calculi, the coal formed during oxidation at high temperatures became a strong absorber itself and the process became self-sustaining.

## Discussion

Infectious and inflammatory complications often occur after operations associated with fine fragmentation of stones (percutaneous nephrolithotripsy, contact ureterolithotripsy), which may be attributed to the release of stone-associated bacteria, biofilms, and their toxins. For example, in the percutaneous nephrolithotripsy group, postoperative infectious and inflammatory complications occurred in 34.1±2.5% of cases (123/361). Extraction of the whole stone led to a decrease in the number of complications (see Figure 2), in the group of percutaneous nephrolithoextraction the rate of complications was 24.6±2.8% of cases (59/240). Dependence on fine fragmentation of the stone and its extraction was also observed during operations on the ureter. For example, complications in the form of inflammatory changes with a temperature reaction were observed in 7.8±1.6% of ureterolithotripsy patients, while this index was 2.5±0.6% in ureterolithoextraction group.

Our studies suggest that one of the possible ways to prevent the development of postoperative complications is to avoid fine stone fragmentation as a source of stone-associated bacterial flora, biofilms, and toxins in the renal system.

It has been shown in studies [5, 6] that the most common postoperative complications after percutaneous puncture nephrolithotripsy with fine fragmentation of stones include development of fever and systemic inflammatory response syndrome (up to 27.4% cases) along with pyelonephritis and problems associated with residual stones. However, this conclusion requires taking into account the cause of body temperature reaction. It is necessary to take into consideration the relationship between the presence of body temperature reaction for 3–4 days after nephrolithoextraction (in our study — in 24.6±2.8% of cases) and the presence of a generalized response to surgical trauma. This was discussed by the authors in the previous study [19]. Thus, an increase in body temperature is possible not only as a response to the action of pyrogens produced in the body mainly by macrophages and neutrophils under the influence of infectious processes, but also pyrogens of non-infectious genesis, such as IL-1, IL-6, TNF-α, TNF-γ — components of acute-phase inflammatory response [20].

At the same time, our data are consistent with the results of similar studies carried out by both domestic and foreign researchers. For example, in a multicenter study by Gadzhiev et al. [21], postoperative fever was observed in 6.3% of patients after percutaneous nephrolithotomy, development of urosepsis was noted in 1.01% of cases.

Infectious and inflammatory complications make the duration of hospital stay longer; the subsequent rehabilitation period increases treatment costs. Thus, the problem of infectious and inflammatory complications after endoscopic surgery, including percutaneous surgical interventions, remains relevant and requires efforts to search for methods of eliminating the associated risk factors. Controlled coarse stone fragmentation was performed by the authors in the bladder as a model for the developing the algorithm of operating procedures. The next step of introducing the method into clinical practice is carrying out such operations in the presence of stones in the renal pelvis. Coarse calculi extraction in patients with infected stones and the presence of a complicated course of urolithiasis can become a method of preventing the development of infectious and inflammatory complications. Moreover, continuous-wave diode lasers required for the procedure are available in any healthcare facility.

## Conclusion

Developed and tested in clinical practice, the method of controlled coarse fragmentation of calculi using a laser with a hot-spot effect in patients with potentially infected stones is promising for prevention of infectious and inflammatory complications as it allows avoiding fine fragmentation of stones and, subsequently, the spread of stone-associated toxins and microflora in the urinary system.
